# A Novel Application of Furazolidone: Anti-Leukemic Activity in Acute Myeloid Leukemia

**DOI:** 10.1371/journal.pone.0072335

**Published:** 2013-08-09

**Authors:** Xueqing Jiang, Lin Sun, Jihui Julia Qiu, Xiujing Sun, Sen Li, Xiyin Wang, Chi Wai Eric So, Shuo Dong

**Affiliations:** 1 Department of Thyroid and Breast Surgery, The Central Hospital of Wuhan, Wuhan, Hubei, China; 2 Department of Medicine and Dan L. Duncan Cancer Center, Baylor College of Medicine, Houston, Texas, United States of America; 3 Department of Pathology and Laboratory Medicine, Temple University School of Medicine, Philadelphia, Pennsylvania, United States of America; 4 Department of Haematological Medicine, King’s College London, Denmark Hill, London, United Kingdom; University of Barcelona, Spain

## Abstract

Acute myeloid leukemia (AML) is the most common malignant myeloid disorder of progenitor cells in myeloid hematopoiesis and exemplifies a genetically heterogeneous disease. The patients with AML also show a heterogeneous response to therapy. Although all-trans retinoic acid (ATRA) has been successfully introduced to treat acute promyelocytic leukemia (APL), it is rather ineffective in non-APL AML. In our present study, 1200 off-patent marketed drugs and natural compounds that have been approved by the Food and Drug Administration (FDA) were screened for anti-leukemia activity using the retrovirus transduction/transformation assay (RTTA). Furazolidone (FZD) was shown to inhibit bone marrow transformation mediated by several leukemia fusion proteins, including AML1-ETO. Furazolidone has been used in the treatment of certain bacterial and protozoan infections in human and animals for more than sixty years. We investigated the anti-leukemic activity of FZD in a series of AML cells. FZD displayed potent antiproliferative properties at submicromolar concentrations and induced apoptosis in AML cell lines. Importantly, FZD treatment of certain AML cells induced myeloid cell differentiation by morphology and flow cytometry for CD11b expression. Furthermore, FZD treatment resulted in increased stability of tumor suppressor p53 protein in AML cells. Our *in vitro* results suggest furazolidone as a novel therapeutic strategy in AML patients.

## Introduction

Acute myeloid leukemia (AML), which is thought to require cooperation between pro-proliferative mutations and defects in myeloid differentiation, is the most common malignant myeloid disorder of progenitor cells in myeloid hematopoiesis [1]. Patients with AML show a heterogeneous response to therapy. The current ‘standard of care’ for AML patients consists of an initial phase of intense chemotherapy (induction) followed by post-remission treatment including additional chemotherapy cycles and/or allogeneic stem cell transplantation. Arabinosylcytosine (Ara-C) and anthracycline- based chemotherapy is the current principal frontline induction therapy for AML. However, treatment responses and outcome for this regimen vary [2], [3]. These therapies usually only result in less than 2 years of median survival and no more than 40% of 5-year overall survival (OS). In recent decades, several promising compounds have been studied in AML, such as Gemcitabine, Paclitaxel, simvastatin and others [3]. The introduction of all-trans retinoic acid (ATRA) to treat acute promyelocytic leukemia (APL), a subtype of acute myeloid leukemia (AML), pioneered a new approach to obtain remission in malignancies by restoring the terminal maturation of leukemia cells [4]. When optimized, combination chemotherapy regimens can offer increased efficacy, decreased toxicity, and even dose reductions. However, ATRA only works well in APL, not in other AML. Also these compounds can elicit serious side effects, such as cardiotoxicity and therapy-related cancer. Therefore, in our study, we aimed to identify a relatively safe and useful compound as a novel treatment for AML patients by screening a library with 1200 FDA approved drugs using the retrovirus transduction/transformation assay (RTTA).

## Results

### Furazolidone can Inhibit the Bone-marrow Transformation Mediated by a Series of Leukemia Fusion Proteins

**Figure 1:**
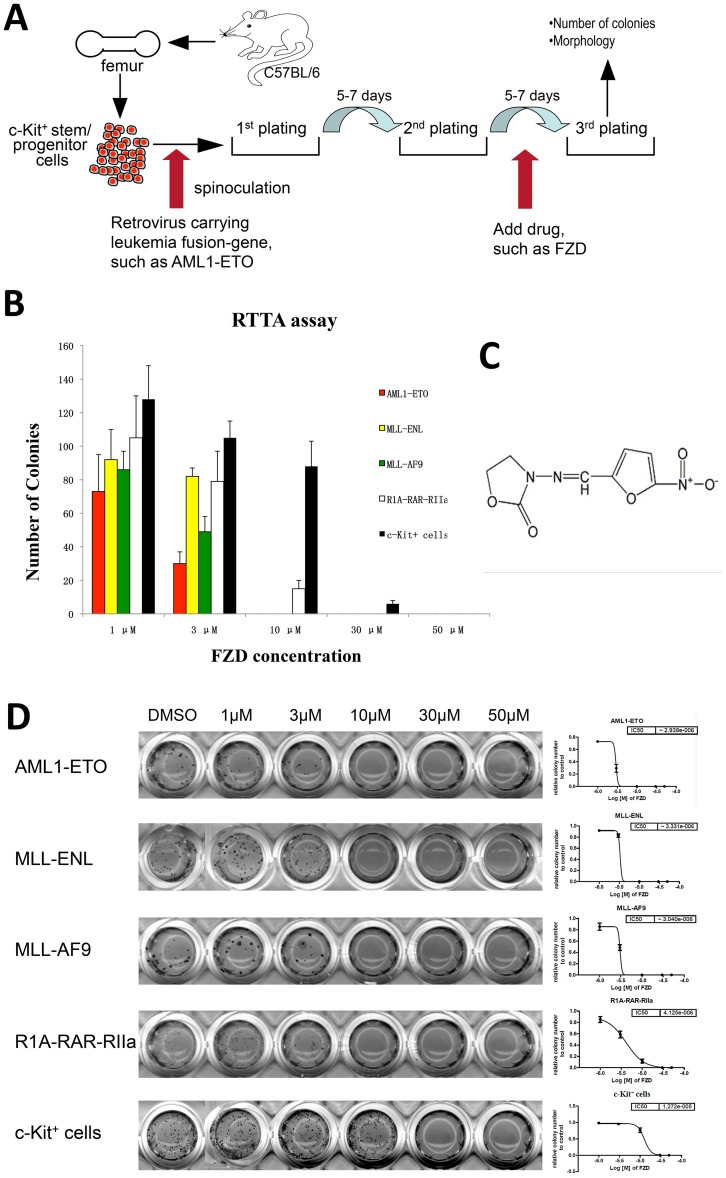
Furazolidone (FZD) suppresses *in vitro* murine bone marrow cell transformation mediated by a series of leukemia fusion proteins. (A) The flow chart of the *in vitro* retrovirus transduction/transformation assay (RTTA) with transduction of leukemia fusions for the initial drug screening. For the initial screening, drugs at 50 µM from the Prestwick chemical library were combined with cells at the 2^nd^ round of plating and added into the methylcellulose medium for the third-round of colony formation. Colonies were counted and morphology was analyzed after 6–7 days and the drugs that had the ability to suppress colony formation of the third-round replating were selected. Next, these selected drugs were tested on c-Kit positive cells at the first-round of plating as a control test for drug toxicity. (B) The bar chart of corresponding absolute colony numbers after the third round of re-plating in methylcellulose culture medium. The concentrations of FZD (from 1 µM to 50 µM) used in bone marrow cells transformed with a panel leukemia fusion genes are indicated. Data are mean ± SD of 3 independent experiments. (C) The structure of FZD. (D) Typical third-round colonies of murine primary bone marrow cells transduced with retroviruses carrying various leukemia fusion genes, after treatment with increasing concentrations of FZD or DMSO control. The graph shows the relative colony numbers after the third-round of re-plating for cells expressing AML1-ETO, MLL-ENL, MLL-AF9, R1A-RAR-RIIa as well as non-transduced c-Kit^+^ murine hematopoietic cells, after treatment with increasing concentrations of FZD. The IC50 values (for inhibition of colony formation by RTTA) are shown in the right panel.

Acute myeloid leukemia (AML) is a hematological malignancy with poor prognosis, and therefore there is a pressing need to develop drugs with excellent activity and fewer toxic side effects [2]. For this purpose, we applied our retrovirus transduction/transformation assay (RTTA) [5], [6], [7], in which leukemic fusion proteins such as AML1-ETO were expressed under the control of long terminal repeat of the murine stem cell virus (MSCV) after transduction into c-Kit^+^ murine hematopoietic progenitor/stem cells [8] (Figure 1A). In RTTA, C57BL/6 murine primary hematopoietic precursor/stem cells positively selected for expression of the progenitor marker c-Kit are transduced with retroviruses carrying a leukemia fusion gene. After serially re-plating in methylcellulose growth medium, we can determine the cellular leukemic transformation potential *in vitro*. In this assay, non-transformed c-Kit^+^ cells form colonies only in the initial plating and are not able to form colonies in the second or third round of plating, because they will rapidly exhaust their self-renewal potential [5], [6], [7]. In contrast, transformed cells can form compact colonies in the third round of plating. In contrast to other knock-in approaches, *in vitro* RTTA assay has offered an alternative solution to study any given oncoproteins involved in leukemic transformation in a much shorter time and more cost-effective manner (Figure 1A).

For the initial screening in our study, the second round transformed cells were screened against a small molecule Prestwick chemical library, which comprises 1200 off-patent marketed drugs and natural compounds that have been approved by FDA in the past for various indications. The cells from the second replating and individual drugs at 50 µM were combined and added into the methylcellulose medium for the third re-plating. The initial screening generated 95 hits out of 1200 compounds (7.9%) with complete or partial suppression of murine bone marrow cell transformation by AML1-ETO (Figure 1A). Next, these 95 compounds were tested for their effects on normal murine bone-marrow c-Kit positive cells. One compound named furazolidone (FZD) (Figure 1B, C) [9] was identified, which showed a higher 50% inhibition concentration (IC50) value of 12.7 µM, compared to 2.9 µM for AML1-ETO transformed cells (Figure 1D).

Furazolidone (N- (5-nitro-2-furfurylidene)-3-amino-2-oxazolidone (Figure 1C)) is a nitrofuran, which has been used for more than sixty years in the treatment of certain bacterial and protozoan infections in human and animals [9], [10]. It has been used empirically with other drugs to treat *Helicobacter pylori (Hp)* infected peptic ulcer disease in China for over 30 years with good result [11]. Therefore, its pharmacokinetics, bioavailability and safety in humans have already been described. In this study, FZD was identified as a potential therapeutic candidate for acute myeloid leukemia (AML)., Therefore, we continued to test the efficacy of FZD on primary cells transformed by other leukemia fusion proteins including MLL-ENL, MLL-AF9, and R1A-RAR-RIIa [5], [6], [7], [8], [12] (Figure 1B, 1D). As shown in Figure 1D, FZD also inhibited cells transformed by MLL-ENL, MLL-AF9 or R1A-RARA-RIIa. The IC50 values for the RTTA assay ranged from 2.9 µM to 4.1 µM (Figure 1D, right panel). These results indicate that FZD inhibits bone marrow transformation mediated by a series of leukemia fusion proteins, and it is relatively safe for use.

### Furazolidone Significantly Inhibits Proliferation of AML Cell Lines

**Figure 2:**
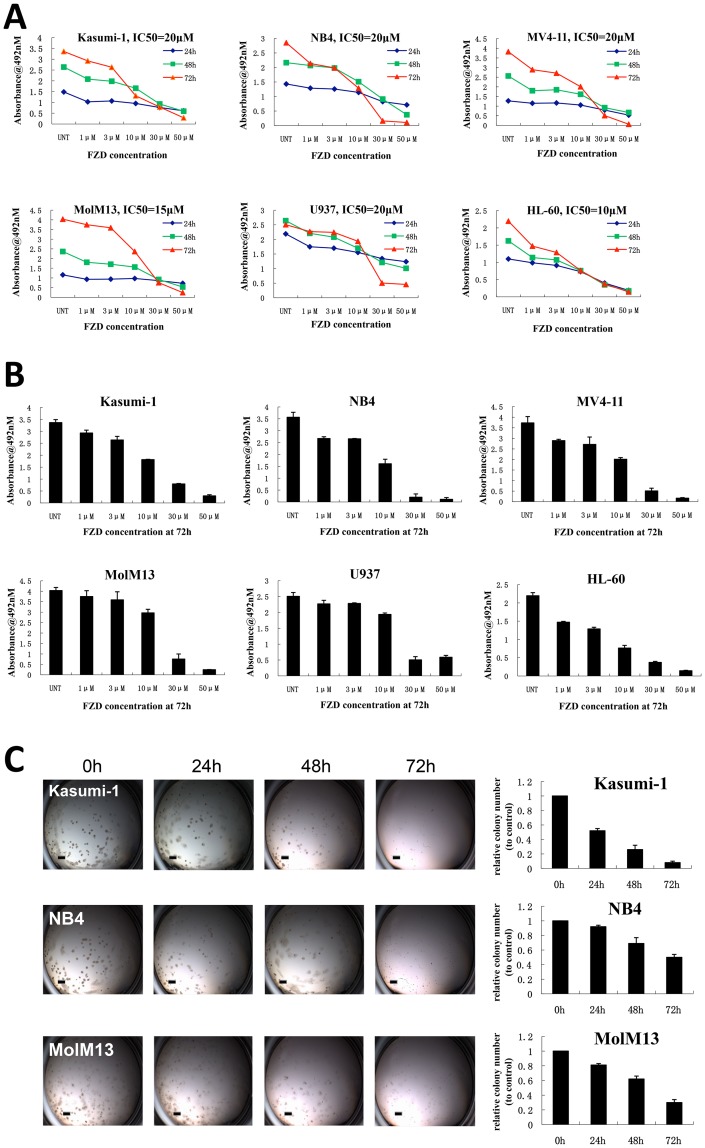
Furazolidone inhibits the proliferation of AML cells. (A) MTS assay in a panel of AML cell lines treated with FZD for 24 h, 48 h and 72 h at indicated concentrations. The data shown are from one representative experiment of three independent experiments. The IC50 values were measured when cells were treated with FZD for 72 hours. (B) MTS assay in AML cell lines after treatment with FZD for 72 hours. Data are mean ± SD of 3 independent experiments. (C) Colony formation assays in Kasumi-1, NB4 and MolM13 cells with DMSO control and FZD treatment at 24, 48, and 72 hours, respectively. Scale bars represent 1 mm. The bar chart represents the relative numbers of colonies in the left panel. Data are mean ± SD of 3 independent experiments.

To further demonstrate the potential anti-proliferation effect of FZD, it was used to treat a panel of AML cell lines (Kasumi-1, NB4, MolM13, MV4-11, U937, and HL-60) with distinct cytogenetic and molecular features (see Table 1). The MTS assay was employed to detect the potential inhibitory effect on cell proliferation after treatment with up to 50 µM FZD for 24, 48, and 72 hours. There was a dose- and time- dependent decrease in viable cell number at all tested concentrations of FZD in cell lines tested. The IC50 ranged from 10 µM to 20 µM (Figure 2A, Figure 2B, Table 1). Furthermore, colony formation assay revealed that the leukemic cells, treated with FZD, had compromised ability to form colonies (Figure 2C). As we know, FZD has been used instead of metronidazole to overcome the high resistance of *Hp.* The usual dose is 100 mg 4 times daily for adults (1.25 mg/kg 4 times daily for children) or 200 mg twice daily for 7 to 14 days [13]. The IC50 values of FZD for induction of DNA damage in human hepatoma G2 cells was reported to be 25–50 µg/ml, which are much higher than the concentrations used in our study [14]. Although there are no reports regarding the anti-tumor or anti-leukemic dose of FZD used in humans, we consider that the IC50 values of 10–20 µM (equal to 2.2–4.5 µg/ml) in our studies should be achievable because the range is comparable to the minimum inhibitory concentration (MIC) for treating *Hp*, which is more than 4 µg/ml [15].

**Table 1:** IC50 values and relevant cytogenetic/molecular data for AML cell lines.

Cell line	Relevant cytogenetic/molecular data	IC50
Kasumi-1	t(8;21)(q22;q22); AML1-ETO; M2-AML	20 uM
NB4	t(15;17)(q22;q11-12); PML-RARα; M3-AML	20 uM
MV4-11	t(4;11)(q21;q23); MLL-AF4; M5-AML	20 uM
MolM13	del(8), ins(11;9)(q23;p22p23); MLL-AF9; M5-AML	15 uM
U937	(Myelo)monocytic; M4/M5-AML	20 uM
HL-60	Myeloblastic; M2-AML	10 uM

### Furazolidone Induces Apoptosis of the AML Leukemic Cells

**Figure 3:**
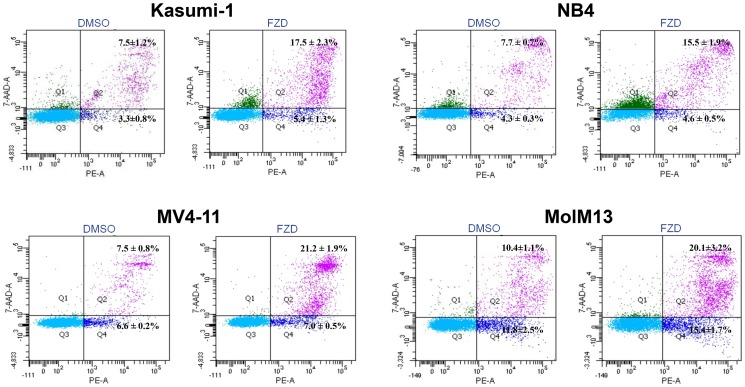
Furazolidone induces apoptosis of AML cells. The apoptosis was measured by Annexin V-PE/7-AAD kit using FACS in Kasumi-1, NB4, MV4-11 and MolM13 in the presence of DMSO control or the predetermined IC50 value of FZD for 72 hours.There are reports that FZD can inhibit cell proliferation and provokes apoptosis by inducing S-phase arrest in human hepatoma G2 cells [14]. We therefore tested whether FZD could also cause cell-cycle arrest in AML cells. We treated AML cells with FZD at the predetermined IC50 value and analyzed by flow cytometry. We showed that this compound did not modulate cell-cycle distribution (Figure S1), suggesting that it prolonged cell-doubling time rather than inducing cell-cycle arrest.

Next, we tested whether FZD could induce apoptosis of these AML leukemic cells. Toward this end, leukemic cells were treated with FZD at the predetermined IC50 value (Table 1) for 72 hours, labeled with Annexin V-PE and 7-AAD, and analyzed by flow cytometry (Figure 3A). Compared with vehicle-treated control (DMSO), FZD was able to induce apoptosis in AML cells: Kasumi-1, 2.1-fold increase in %Annexin V+/7-AAD+ (*P*<0.05); NB4, 1.7-fold increase (*P*<0.05); MV4-11, 2.0-fold increase (*P*<0.05); MolM13, 1.6-fold increase (*P*<0.05) (Figure 3A). However, there was no evidence of increased apoptosis in U937 and HL-60 leukemic cells (data not shown).

### Treatment with Furazolidone Induces Differentiation of AML Cell Lines

**Figure 4:**
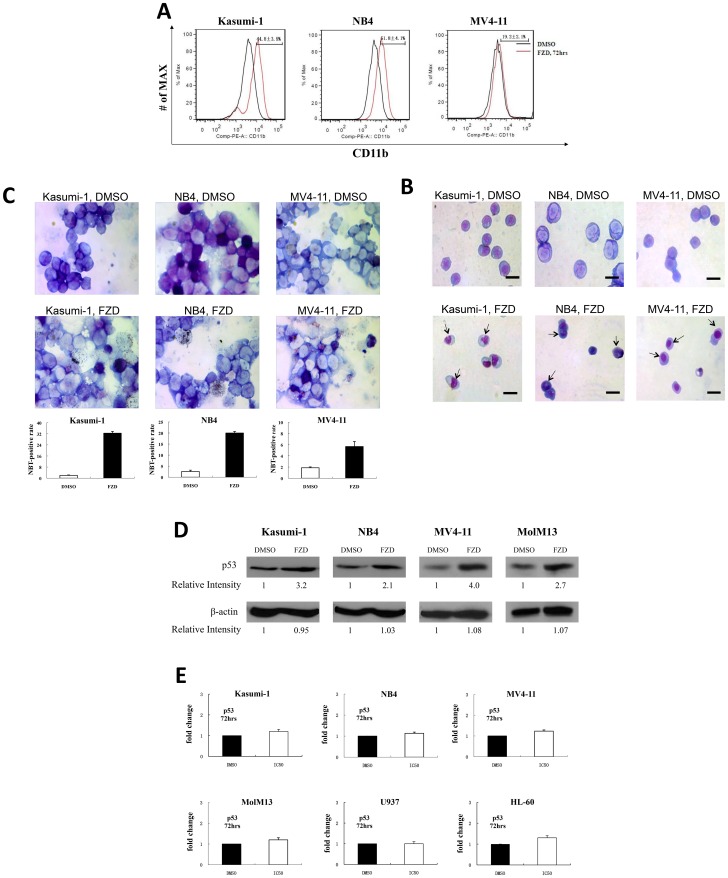
Furazolidone induces the leukemic cellular differentiation. (A) The myeloid differentiation antigen CD11b, was measured by FACS in Kasumi-1, NB4 and MV4-11 after FZD (predetermined concentration) and DMSO control treatment for 72 hours. The representative graph was from one of the three independent experiments. (B) The Giemsa staining of Kasumi-1, NB4 and MV4-11 cells treated with FZD (predetermined concentration) or DMSO control for 96 hours (magnification, 400×). Arrows indicate nuclear condensation and multilobulated nucleus. Scale bars represent 10 µm. (C) Photomicrographs and the relative histograms (bottom panel) of the NBT reduction assay with Kasumi-1, NB4 and MV4-11 cells in the DMSO control group (top panel) and FZD treatment group (middle panel) for 72 hours (Giemsa stain; magnification, 1,000×). There were many black particles (formazan) in the FZD treatment group of leukemia cells, so-called NBT-positive cells, which indicated that the AML cells had differentiated. Data are mean ± SD of 3 independent experiments (bottom panel). (D) The expression of p53 protein was measured by Western blot in Kasumi-1, NB4, MV4-11 and MolM13 cells after 72 hours treatment with the predetermined IC50 value for FZD or control (DMSO). Relative protein levels, normalized to the beta-actin control, were quantitated and listed below each panel. (E) The p53 mRNA expression was measured by RT-PCR in AML cell lines.

Following the successful clinical application of all-trans retinoic acid (ATRA) in the treatment of APL patients by induction of differentiation [1], [4], we investigated whether FZD could induce AML cell differentiation. Indeed, following the treatment of FZD, we observed varying degrees of incremental expression of the myeloid differentiation marker CD11b in Kasumi-1, NB4 and MV4-11 (Figure 4A), but not U937 (date not shown). In addition, we also found the evident morphologic changes characteristic of differentiation, such as the appearance of granules and condensation of the nucleus, by Giemsa staining of FZD-treated cells (Figure 4B).

Next, we also tested the FZD-induced leukemic cell differentiation effect by NBT reduction assay, which is often used to reflect the differentiation of leukemia cells functionally. As expected, the NBT-positive rate of Kasumi-1 and NB4 cells, but not MV4-11 cell, in the FZD treatment groups were significantly higher than those in the DMSO control groups, respectively (*P*<0.05) (Figure. 4C). In our current study, we demonstrated that treatment with FZD in a series of leukemia cells induced both cell apoptosis and differentiation; however the predominant effect in tested leukemic cells is not identical. The effect of differentiation in Kasumi-1 and NB4 is predominant but the apoptosis effect is more obvious in MV4-11 cells.

We have shown that FZD significantly inhibits proliferation, induces apoptosis and differentiation in human AML cell lines; however, the underlined mechanism or the target involved is unknown. As we know, p53 can induce cell apoptosis and it also is a potent mediator of myeloid leukemia cell differentiation [16]. The molecular mechanisms for p53-mediated differentiation are not clear, but seems not to rely on induction of p21 [17] or on cell cycle arrest [18]. We demonstrated that the protein level (Figure 4D), but not mRNA level (Figure 4E), of p53, which is considered a tumor-suppressor, was up-regulated following treatment with FZD in Kasumi-1, NB4, MV4-11 and MolM13 cells. Although we observed a significant increase in p21 mRNA level (Figure S2A, *P*<0.05 in all the cell lines tested), whether it has any impact on the anti-leukemic effects of FZD is still unknown. We therefore knocked down endogenous p21 expression using shRNA in NB4 cells. We then treated these cells with FZD at the predetermined IC50 concentration or DMSO for 72 hours, and measured proliferation over time. As shown in Figure S2B and Figure S2C, FZD-mediated inhibition of proliferation is not different in p21 down-regulated NB4 cells compared to parental NB4 cells. The anti-leukemia activities of FZD may be attributed to its effect on p53 stability, which needs to be further studied in future.

## Discussion

In this study, we investigated the anti-leukemic activity of a chemical library containing more than 1000 FDA approved drugs and identified furazolidone (FZD) as a potent inhibitor that suppressed the self-renewal of murine bone marrow cells transformed by AML1-ETO. FZD has been used for more than sixty years but there are no reports about the anti-leukemia effect of this drug. In the current study, we have shown the ability of this drug to inhibit proliferation and induce apoptosis and differentiation in human leukemia cells. In our study, FZD had no significant impact on cell-cycle, which is different from one recent study, in which FZD could inhibit human hepatoma cell proliferation and increased apoptosis by inducing S-phase cell cycle arrest [14].

The disruption of terminal differentiation is a salient feature in the pathogenesis of AML, and differentiation-based anticancer treatments, such as all-*trans* retinoic acid (ATRA), have been developed to overcome this block, thereby leading to clinical remission of APL patients [4], [19]. Here, we showed that treatment of AML cancer cells with FZD induced leukemic cell differentiation, evident by the increased expression of the myeloid differentiation marker CD11b, NBT reduction assay, as well as morphologic changes, specifically in Kasumi-1, carrying a t(8; 21) translocation [8]. In acute myeloid leukemia (AML), the (8; 21) translocation that generates AML1-ETO fusion gene is associated with about 40% of AML-M2 cases and represents the most frequent chromosomal anomaly [20], [21]. However, therapy for this subtype of AML is limited as there is not yet an efficacious, targeted drug like ATRA. In our study, these anti-leukemia effects could be attributed to the up-regulation of p53 protein expression, which is well known to mediate leukemia cell differentiation through a p21-independent pathway [17].

Furazolidone has been used for more than sixty years in the treatment of certain bacterial and protozoan infections in human and animals [9]. It was also used as feed additives for many years, but was found to be mutagenic and genotoxic [22]. Despite a diminution in its use in the last three decades, FZD is available for medical and veterinary use for the treatment of cholera, bacterial diarrhea and giardiasis [10]. Rabbani and colleagues conducted a randomized double-blind placebo-controlled trial in children with cholera and found that this compound was clinically effective and no patient experienced significant toxicity [23]. FZD, as a synthetic nitrofuran antimicrobial drug, has broad antibacterial activity based on interference with bacterial enzymes. It was reported that it also has activity against H. pylori and can be used to replace metronidazole as a rescue therapy [24]. It is a very popular antibiotic in China recently. FZD has been shown to be an inhibitor of monoamine oxidase (MAO) activity in the tissues of animals. The drug itself is not a MAO inhibitor, and must be transformed *in vivo* to metabolites that can inhibit the enzyme activity [9].

Taken together, here we report the biologic and pharmacologic activity of FZD in a series of AML cells, in which FZD has potent anti-leukemic activities. Our data are the first to suggest a new clinical therapeutic application of furazolidone in acute myeloid leukemia (AML), especially AML1-ETO positive AML.

## Materials and Methods

### Cell Culture and Reagent

AML cell lines, NB4 and U937, were described previously [5], [25]. Kasumi-1, MV4-11 and HL-60 leukemic cells were purchased from ATCC (American Type Culture Collection, Manassas, VA), while MolM-13 was obtained from DSMZ (Deutsche Sammlung von Mikroorganismen und Zellkulturen GmbH, Braunschweig, Germany). These leukemic cells were cultured in RPMI 1640 medium (Life Technologies, Carlsbad, CA) supplemented with 10% FBS (NB4, MV4-11, MOLM-13, U937 and HL-60) or 20% FBS (Kasumi-1) (Life Technologies, Carlsbad, CA). GP2-293 cells (Clontech Laboratories, Mountain View, CA) and HEK293T cells were cultured in Dulbecco modified Eagle medium (DMEM) with 10% FBS (Life Technologies, Carlsbad, CA) [6]. Furazolidone (FZD) was purchased from Sigma-Aldrich (Sigma-Aldrich, St. Louis, MO).

### RTTA Assay

Retrovirus transduction/transformation assay (RTTA) was performed as described previously [5], [6], [7], [8]. Briefly, the c-Kit positive cells were isolated from murine bone marrow hematopoietic progenitor/stems cells by magnetic activated cell sorting (MACS; Miltenyi Biotec, Bergisch Gladbach, Germany). The animal studies were approved by the Institutional Animal Care and Use Committee of Baylor College of Medicine. The viral supernatants were collected 48 to 72 hours after transfection of GP2-293 cells and were used to infect the c-Kit^+^ cells. Transduced bone marrow cells were plated in 1% myeloid-conditioned methylcellulose, which contained an Iscove modified Dulbecco medium–based Methocult (Methocult M3231; StemCell Technologies, Vancouver, Canada) and supplemented with 20 ng/mL of recombinant murine SCF, 10 ng/mL each of IL-3, IL-6, and granulocyte macrophage colony-stimulating factor (GM-CSF) (PeproTech, Rocky Hill, NJ). Replating was repeated every 5-7 days. The test compounds from the Prestwick chemical library (Prestwick Chemicals, France) were mixed well with the second-round replating cells, and subsequently added to 48-well plate at a final concentration of 50 µM. For drug studies, furazolidone (FZD) was diluted in DMSO and added to the transduced cells before the third plating. Transformation results were determined from at least 3 independent experiments.

### Cell Viability Assays

Leukemic cells were seeded in 96-well culture plates at a density of 1 or 2×10^4^ viable cells/100 µl/well in triplicates and were treated for 24, 48, and 72 hours with an incremental concentration of FZD ranging from 1 µM to 50 µM. Colorimetric CellTiter 96® Aqueous One Solution Cell Proliferation assay (MTS assay; Promega, Madison, WI) was used to determine the cytotoxicity. The optical density at 492 nm was measured using a Multiskan Ascent® microplate photometer (Thermo Fisher scientific, Waltham, MA). IC50 values were determined by MTS assay when cells were treated with FZD for 72 hours and calculated with GraphPad Prism 5. Each experiment was in triplicate.

### Colony Formation Assay

The leukemic cells (Kasumi-1, NB4 and MolM-13) were treated with furazolidone for 24, 48 and 72 hours, respectively, after which they were washed with PBS at least three times. Cells were resuspended in RPMI-1640 medium without FBS. Approximately 600 viable cells were mixed with Methocult medium (Methocult H4100; StemCell Technologies, Vancouver, Canada) and plated into a 24-well plate. Colonies were counted under a light microscope after 14 days.

### Apoptosis Assay

The leukemic cells were treated with FZD at the IC50 concentration for 72 hours. Apoptosis was assessed using Annexin V-PE/7-AAD Kit following the manufacturer’s instructions (Becton Dickinson, Franklin Lakes, NJ) [5]. Briefly, the harvested cells were washed at least once with PBS (with Ca^2+^ and Mg^2+^), resuspended in Binding Buffer, and then transferred to a polystyrene round bottom test tube (Becton Dickinson, Franklin Lakes, NJ). Then, PE Annexin V and 7-AAD were added into each sample and incubated for 15 min at room temperature in the dark. After that, the analysis was done by flow cytometry with a BD LSR Fortessa, and the data analysis was performed using FlowJo Version 7.6. Results are representative of three independent experiments performed in duplicate.

### Flow Cytometry Assay

Cell lines Kasumi-1, NB4 and MV4-11 were treated with DMSO or FZD at the predetermined IC50 concentration for 72 hours. A density of 1×10^5^ viable cells were harvested, washed with PBS and then stained with CD11b (clone M1/70) PE antibody (Biolegend, San Diego, CA). The cells were incubated for 10 min at 4°C in the dark, washed with PBS and analyzed on LSR II (BD Biosciences, Franklin Lakes, NJ). Data analysis was performed using FlowJo Version 7.6. Results are representative of 3 independent experiments performed in duplicate.

### Morphologic Examination

The leukemic cells were treated with FZD or DMSO for 4 days, harvested, washed in PBS and then 4×10^4^ viable cells were prepared for cytospin onto glass slides (5 min spin at 500 rpm). The cells on glass slides were stained with Giemsa (WG16; Sigma-Aldrich, St. Louis, MO) for 5 minutes, rinsed briefly with distilled water, dried, and then observed under microscopy [6].

### NBT Reduction Assay

For NBT reduction assay, the cells were harvested, added to 0.5 ml NBT reaction solution (1 mg/ml NBT and 100 ng/ml TPA) (Sigma-Aldrich, St. Louis, MO), and incubated at 37°C for 1 hour. The cell sediments were spread on glass slides, stained with Giemsa solution, and examined under oil immersion objective. The percent of NBT-positive cells was determined from counting at least 200 total cells. The test was performed three times and the results were expressed as a mean of triplicates: NBT-positive cell rates = NBT-positive cell counts/total cell counts × 100%.

### Generation of p21-knockdown NB4 Cells

The pGIPZ lentiviral vectors carrying CDKN1A (p21)-short hairpin RNA (shRNA) and Scrambled-shRNA (non-silencing) were purchased from Cell Based Assay Screening Service of Baylor College of Medicine. The nucleotide sequence of two shRNA targeting CDKN1A (p21) was: (1) 5′-CAGCCTCTGGCATTAGAAT-3′ and (2) 5′- CTGATCTTCTCCAAGAGGA-3′. HEK293T cells were transfected with packaging mix and short hairpin RNA (shRNA) vector DNAs. After 48–72 hours of transfection, the supernatant was collected, concentrated, and viral titer determined. Viral supernatant was used to infect NB4 leukemia cells, and stably transduced cells constitutively repressing p21 were selected in the presence of 2 µg/ml puromycin. Knockdown of p21 was confirmed by RT-PCR.

### Real-time Quantitative Reverse Transcription-PCR and Western Blot

The Real-time Quantitative Reverse Transcription-PCR analysis and Western blot analysis were performed as described in our previous studies [26].

### Cell-cycle Analysis

For cell-cycle analysis, the cells were treated with the indicated concentration of FZD for 24 hours, and fixed in 70% ice-cold ethanol overnight at −20°C. Before analysis, cells were washed with PBS, stained with DNA staining solution which contained 200 µg/ml of RNaseA (Sigma-Aldrich, St. Louis, MO) and 50 µg/ml of PI (Sigma-Aldrich, St. Louis, MO) and transferred to a 5 ml polystyrene tube with filter to remove cell clumps. Then cells were incubated for 30 min at 20°C protected from light, and kept on ice until the analysis by flow cytometry with a BD Cantoll (BD Biosciences, Franklin Lakes, NJ). Data analysis was performed using FlowJo Version 7.6. Results are representative of three independent experiments performed in duplicate.

### Statistical Analysis

The results were expressed as means ± SD. The differences between Furazolidone-treated and control cells were analyzed using *t* test. The 2-sides value of *P*<0.05 was considered statistically significant.

## Supporting Information

Figure S1 **FZD has no effects on cell-cycle in AML cells.** Cell-cycle assessment in the tested acute myeloid leukemic cell lines using propidium iodine (PI) detected by flow cytometry at 24 hours.
(TIF)

Figure S2 **p21 is dispensable in FZD-mediated inhibition of NB4 cell proliferation.** (A) The p21 mRNA expression was measured by RT-PCR in AML cell lines after 72 hours treatment with the predetermined IC50 value for FZD treatment or control (DMSO). (B) Knockdown of p21 expression in NB4 cells was assayed using real time PCR. (C) MTS assay was used to measure the proliferation after treatment of FZD at the predetermined IC50 value in the p21-knockdown stable NB4 cell line and control cells. Data are mean ± SD of 3 independent experiments.
(TIF)
