# Supplementary figures and images for: A Novel Application of Furazolidone: Anti-Leukemic Activity in Acute Myeloid Leukemia
Source: PLoS One. 2013 Aug 9;8(8):e72335. doi: 10.1371/journal.pone.0072335 (PMC3739762; doi:10.1371/journal.pone.0072335)

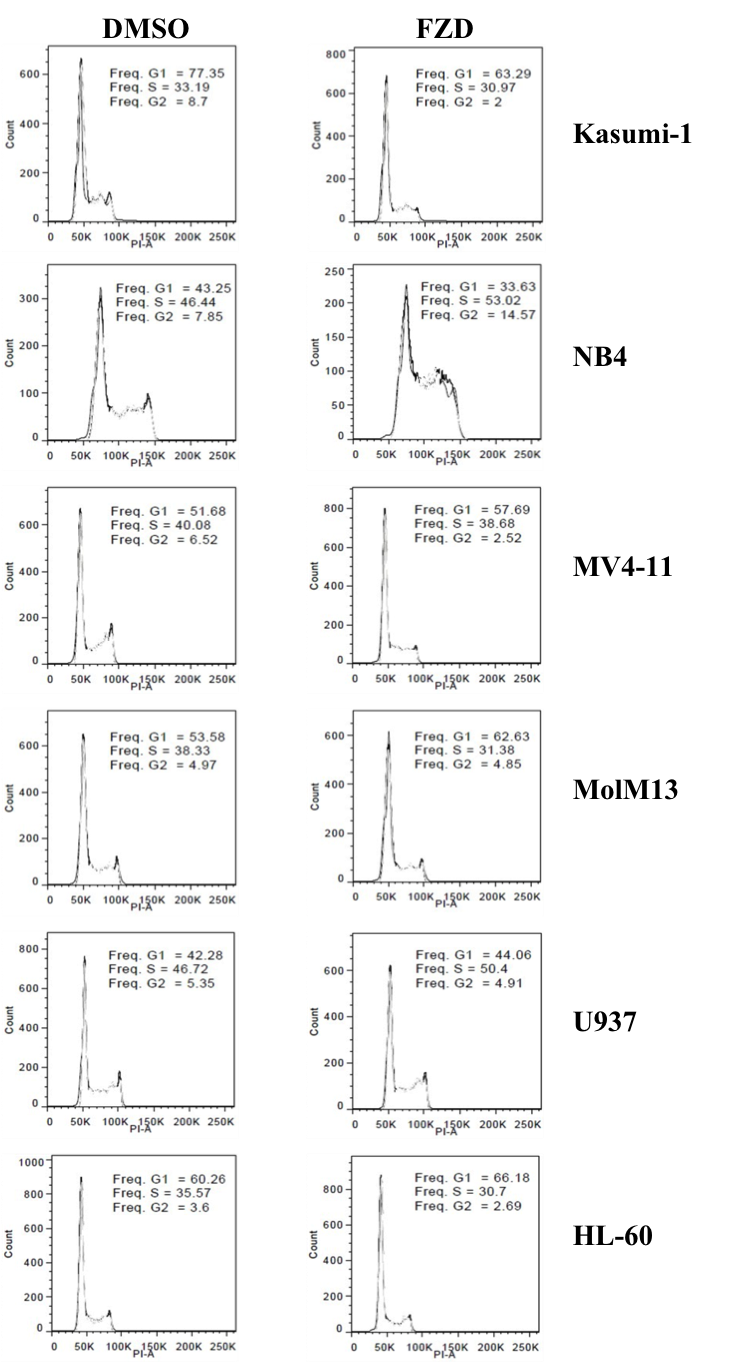

Supplement: Figure S1 — FZD has no effects on cell-cycle in AML cells. Cell-cycle assessment in the tested acute myeloid leukemic cell lines using propidium iodine (PI) detected by flow cytometry at 24 hours. (TIF) [file pone.0072335.s001.tif]

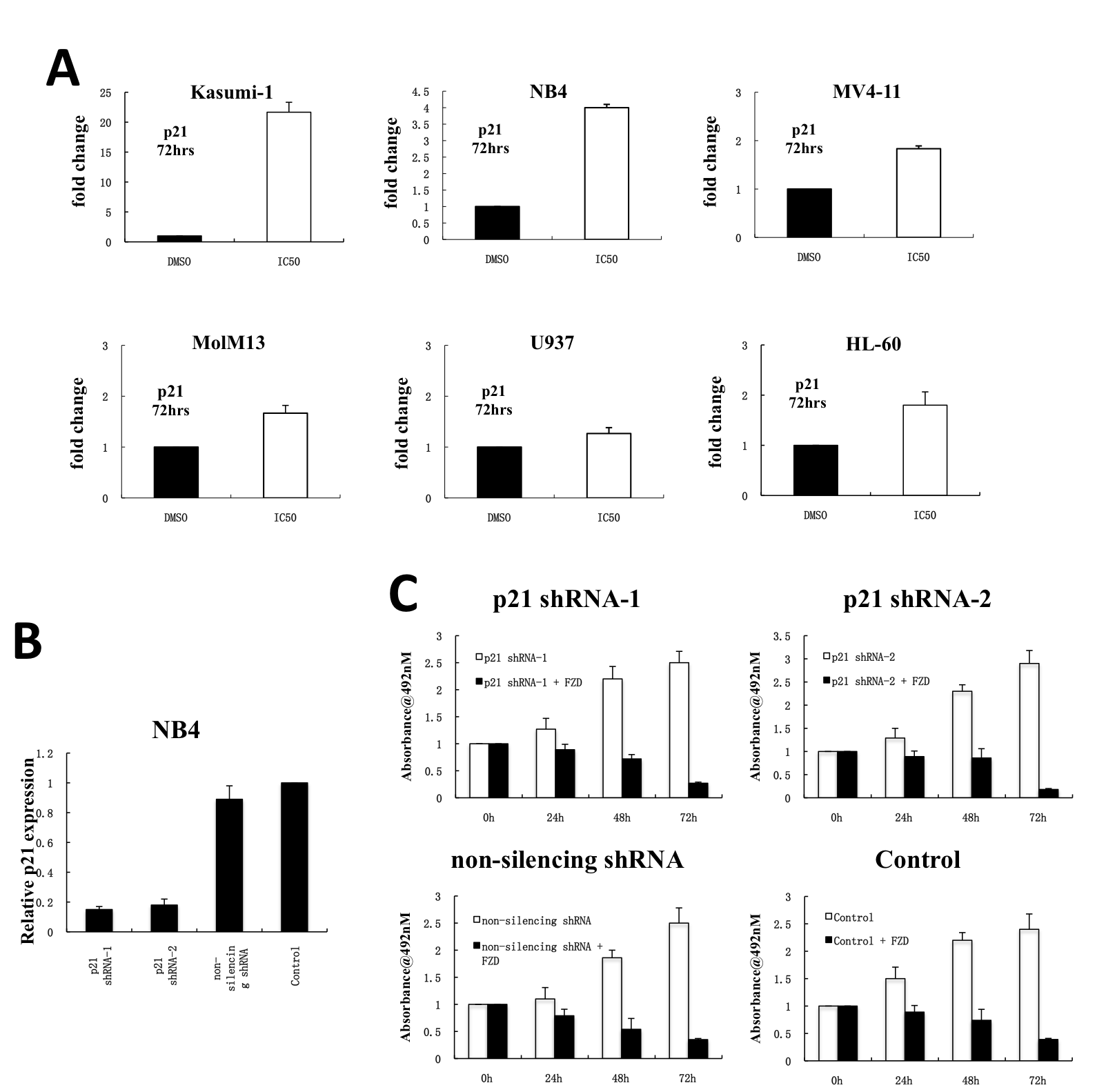

Supplement: Figure S2 — p21 is dispensable in FZD-mediated inhibition of NB4 cell proliferation. (A) The p21 mRNA expression was measured by RT-PCR in AML cell lines after 72 hours treatment with the predetermined IC50 value for FZD treatment or control (DMSO). (B) Knockdown of p21 expression in NB4 cells was assayed using real time PCR. (C) MTS assay was used to measure the proliferation after treatment of FZD at the predetermined IC50 value in the p21-knockdown stable NB4 cell line and control cells. Data are mean ± SD of 3 independent experiments. (TIF) [file pone.0072335.s002.tif]
